# Cauli-Det: enhancing cauliflower disease detection with modified YOLOv8

**DOI:** 10.3389/fpls.2024.1373590

**Published:** 2024-04-18

**Authors:** Md. Sazid Uddin, Md. Khairul Alam Mazumder, Afrina Jannat Prity, M. F. Mridha, Sultan Alfarhood, Mejdl Safran, Dunren Che

**Affiliations:** ^1^ Department of Computer Science, American International University-Bangladesh, Dhaka, Bangladesh; ^2^ Department of Computer Science, College of Computer and Information Sciences, King Saud University, Riyadh, Saudi Arabia; ^3^ School of Computing, Southern Illinois University, Carbondale, IL, United States

**Keywords:** cauliflower disease detection, machine vision, YOLOv8, agricultural disease management, vegetable disease detection

## Abstract

Cauliflower cultivation plays a pivotal role in the Indian Subcontinent’s winter cropping landscape, contributing significantly to both agricultural output, economy and public health. However, the susceptibility of cauliflower crops to various diseases poses a threat to productivity and quality. This paper presents a novel machine vision approach employing a modified YOLOv8 model called Cauli-Det for automatic classification and localization of cauliflower diseases. The proposed system utilizes images captured through smartphones and hand-held devices, employing a finetuned pre-trained YOLOv8 architecture for disease-affected region detection and extracting spatial features for disease localization and classification. Three common cauliflower diseases, namely ‘Bacterial Soft Rot’, ‘Downey Mildew’ and ‘Black Rot’ are identified in a dataset of 656 images. Evaluation of different modification and training methods reveals the proposed custom YOLOv8 model achieves a precision, recall and mean average precision (mAP) of 93.2%, 82.6% and 91.1% on the test dataset respectively, showcasing the potential of this technology to empower cauliflower farmers with a timely and efficient tool for disease management, thereby enhancing overall agricultural productivity and sustainability

## 1 Introduction

Agriculture is not only the primary source of food security, in agriculturally driven countries like Bangladesh, it is also one of the main sources of employment opportunities (Eunice et al., 2022). The agricultural sector holds a crucial position in maintaining rural communities, particularly in developing nations, as it provides the primary source of sustenance, income, and employment. This sector contributes around 6.4% of global economic productivity which surmounts to over 5 million dollars (Statistics Times, 2018). Bangladesh is considered an agricultural nation, and its economy relies significantly on agricultural production. According to The Global Economy, up until 2022, more than 37% of the workforce is engaged in agriculture (The Global Economy, 2021) and accounts for 11.50% of the GDP of the nation (Bangladesh Bureau of Statistics, 2023). In Bangladesh, where agriculture is crucial, Cauliflower is a notable vegetable appreciated both for its popularity, health benefits and economic significance. Cauliflower belongs to the Brassicaceae family and is full of fiber and vitamins B (Pourdarbani and Sabzi, 2023), which are beneficial to health. Being a cruciferous vegetable, it provides heart-healthy fiber and choline, a substance critical for learning, memory, muscles, and sleep. Cauliflower is incredibly versatile in the kitchen, fitting seamlessly into various dishes whether consumed fresh or cooked.

Cauliflower is cultivated in numerous countries. In terms of production volume, the top countries include China, India, USA, Mexico, Spain, Italy, Turkey and Bangladesh. Bangladesh produced 283 kilotons of cauliflower in 2020 (Helgi Library, 2022). The ideal growing conditions for cauliflower are areas between 11 and 60° N with typical temperatures ranging from 5 to 28°C. While developing, it can tolerate temperatures ranging from -10°C to 40°C for a few days (Singh et al., 2018). Cauliflower farming faces challenges from several diseases like bacterial spot rot, black rot, downy mildew etc. The growth and productivity of cauliflower can be significantly impacted by these diseases. Farmers must identify these diseases early on and use the right method to control them. There exists multiple approaches for the control of cauliflower diseases such as physical control (hot water, nanoparticles), chemical control (pesticides) and biological control (Aqueous extracts, Bacillus etc.) (Liu et al., 2022). Pesticides used to protect cauliflower can be harmful to human health, and diseases caused by bacteria or fungus can cause problems including allergies when consumed (Pathak et al., 2022). This has an impact on the amount and quality of cauliflower as well as contributing to the annual loss of a significant chunk of harvests to plant diseases. Traditional ways of identifying cauliflower diseases in farming face significant challenges. Frequently, they depend on manual inspection, which is laborious, error-prone, and can miss early diseases. Most farmers, especially those in remote areas, cannot afford to hire and retain agricultural experts for disease identification.

The increasing adoption of optical imaging, machine vision and artificial intelligence techniques in vegetable disease detection and management has resulted in increasing demand for these applications in various areas of precision agriculture (Teet and Hashim, 2023). Convolutional Neural Network (CNN) approaches, in particular, are a promising option offered by more recent techniques utilizing modern technologies (Gu et al., 2018). These methods are faster, more accurate, and scalable, allowing for continuous crop monitoring. Many researchers are actively involved in identifying cauliflower diseases using CNN approaches, but these methods, while effective in disease classification, fall short in the problem of disease localization. In our research, we analyzed the latest methods for detecting and classifying crop diseases (especially cauliflower diseases) and introduced an approach using the YOLOv8 object detection model as a base which not only classifies cauliflower diseases but also identifies the region of affected areas in the images. Among all diseases, we worked on Black Rot, Downy Mildew, and Bacterial Sport Rot.

This paper introduces a modified YOLOv8 model for the localization and labelling of cauliflower diseases. This model combines the pre-trained knowledge of the YOLOv8 model with extra convolutional layers to improve the accuracy for identifying cauliflower disease. The primary contributions of this paper are enumerated as follows:

1. Custom YOLOv8s Model Development: The paper introduces a tailored YOLOv8s model designed specifically for identifying three prevalent cauliflower diseases—Downey mildew, bacterial spot rot, and black rot. The development of this custom model addresses the unique challenges posed by cauliflower diseases.
2. Performance Evaluation of Base YOLOv8 Models: The study conducts a comprehensive evaluation of base YOLOv8 models on a cauliflower disease detection dataset. By employing rigorous testing and comparison methodologies, the paper sheds light on the baseline performance of YOLOv8 models in the context of cauliflower disease detection.
3. Systematic Model Modifications for Improved Detection Performance: Building upon the baseline evaluation, the paper systematically applies different modifications to enhance the detection accuracy and average precision of the base YOLOv8 model. This contributes valuable insights into the specific adjustments and fine-tuning strategies that yield improvements in the model’s ability to accurately detect and classify cauliflower diseases.
4. Open Access to Annotated Dataset and Proposed Model: The paper not only presents novel insights into custom model development and systematic modifications but also contributes to the research community by providing an annotated version of the VegNet cauliflower disease classification dataset (Sara et al., 2022). This dataset, along with the proposed custom YOLOv8s model, is made openly accessible. This contribution facilitates reproducibility, encourages further research, and establishes a foundation for ongoing advancements in the field of computer vision applied to agriculture.

The remaining portions of the paper are organized as follows: We discuss the existing literature for cauliflower disease detection and related problems in Section 2. We talk about an overview of the dataset and generally used image pre-processing techniques, and the methodology of the paper is explained in Section 3. We present the results of the performance observation of the models in Section 4. We discuss our findings in Section 5. The conclusion and potential future research endeavors are discussed in Section 6.

## 2 Literature review

Crop diseases pose serious risks to food production, economic stability, and food security, having a substantial impact on global agriculture. These diseases have the potential to cause significant yield losses, endangering the livelihoods of millions of farmers and putting vital crops needed for commerce and subsistence at risk. In addition to being essential for maintaining a steady and secure food supply, crop diseases highlight the need for novel approaches, like the use of cutting-edge technologies like deep learning, to improve disease detection and mitigation techniques in the agricultural industry. The most recent and notable research endeavors in this domain are discussed in this section.

(Arun and Umamaheswari, 2023) introduced the Complete Concatenated Deep Learning (CCDL) framework, a multiple crop disease classification model capable of labelling crop diseases across many species of crop. The core functional unit of this architecture is the Complete Concatenated Block (CCB), which strategically places a point-wise convolution layer before each convolution layer to limit the increment of parameters in the model. The reorganized Plant Village dataset was used by the researchers to train this architecture. The PCCDL-PSCT approach proposed by the authors performed best, obtaining an impressive accuracy of 98.14% with a smaller model size of about 10 MB. (Chug et al., 2023) presented an innovative framework that combines the strengths of both machine learning and deep learning. The proposed framework comprises 40 diverse Hybrid Deep Learning (HDL) models. The performance of the HDL models was notably impressive on the IARI-TomEBD dataset, achieving high accuracy levels ranging from 87.55% to 100%. To validate the effectiveness of the approach, the researchers conducted experiments using two publicly available plant disease datasets, namely PlantVillage-TomEBD and PlantVillage-BBLS. (Huang et al., 2023) introduced an approach based on FC-SNDPN (Fully Convolutional – Switchable Normalization Dual Path Networks) for the automated detection and identification of crop leaf diseases. To mitigate the impact of complex backgrounds on the recognition of crop diseases and insect pests, the authors utilized a Full Convolutional Network (FCN) algorithm based on the VGG-16 model for target crop image segmentation. The SNDPN approach unites the connection method between DenseNet and ResNet layers, forming a neural network utilizing Switchable Normalization (SN) layers. The method proposed combines SNDPN for detecting diseases and FCN for segmenting the foreground, demonstrated an identification accuracy of 97.59% on the augmented dataset, affirming the efficacy of the proposed methodology. (Haridasan et al., 2023) employed a computer vision-centric approach, incorporating image processing, ML and DL techniques to diminish reliance on traditional methods for safeguarding paddy crops against diseases. The utilization of image segmentation to pinpoint the afflicted regions of the paddy plant was proposed, identifying diseases solely based on their visual characteristics. A combination of a SVM classifier and CNN was employed for the recognition and classification of specific types of paddy diseases. By incorporating ReLU and SoftMax functions, the proposed deep learning strategy achieved a validation accuracy of 91.45%.

(Mallick et al., 2023) presented an innovative deep learning approach for the identification of pests and diseases affecting mung beans. To address the challenge posed by the limited number of available mung bean crop images available for training, the researchers employed transfer learning, which yielded highly promising results for swift and effective disease as well as pest detection. The proposed model successfully distinguished 6 types of mung bean diseases and 4 types of pests from healthy and diseased leaves collected across various seasons. Through experimentation, the proposed lightweight DL model for mung bean disease and pest detection demonstrated an impressive average accuracy of 93.65%. (Zhao et al., 2023) introduced enhancements to the YOLOv5s model for improved crop disease detection. The modifications included refining the CSP structure in the feature fusion stage, incorporating a lightweight composition to reduce model parameters, and extracting feature information through multiple branches. Addressing scaling issues during training, an improved DIoU loss function replaced the Generalized IoU loss function from the original YOLOv5. Through transfer learning, the enhanced model exhibited superior mean average precision (mAP) compared to YOLOv3, YOLOv4, YOLOv4-tiny, YOLOv5s, Faster R-CNN and SSD models, achieving recall, F1 and mAP mAP, F1 score, and recall of 87.89%, 91%, and 95.92%, respectively. These values marked improvements of 4.58%, 5%, and 4.78%, respectively, compared to YOLOv5s. (Lin et al., 2023) introduced an enhanced YOLOX-Tiny network, denoted as YOLO-Tobacco, designed for detecting brown spot disease in open-field tobacco crop images. Their objective was to uncover crucial disease features and improve the fusion of diverse feature levels, facilitating the detection of dense disease spots across various scales. The YOLO-Tobacco network demonstrated an AP (average precision) of 80.56% on the test set, surpassing available lightweight detection models such as YOLOX-Tiny, YOLOv5s, and YOLOv4-Tiny by 3.22%, 8.99%, and 12.03%, respectively. (Hu et al., 2023a) introduced a novel Multi-Scale Dual-branch model for pest identification from rice crop images, employing a GAN (generative adversarial network) and an enhanced ResNet to discern pests in complex background images. To optimize the calculations ratio of residual blocks, the ConvNeXt residual block was incorporated into the ResNet model and a dual-branch structure was devised to extract features of disease affected spots of varying sizes, adjusting the convolution kernel size for each branch. Training the new model on a systematically expanded dataset improved recognition accuracy by 2.66% compared to the original ResNet model. In comparison with base networks like AlexNet, DenseNet, VGG, ResNet, and Transformer under similar conditions, the new model demonstrated superior performance, achieving a disease recognition accuracy of 99.34%.

(Thakur et al., 2023) presented a lightweight CNN model named ‘VGG-ICNN’ designed for identifying crop diseases through plant-leaf images. The VGG-ICNN model comprises approximately 6 million parameters, significantly fewer than many existing high-performing DL models. The model’s effectiveness was assessed across five diverse public datasets encompassing various crop types, including multi-crop datasets like Embrapa and PlantVillage with 93 and 38 categories, respectively, and single crop datasets like Maize, Rice and Apple each with four or five categories. Experimental outcomes indicated that the proposed method surpassed several recent DL approaches in crop disease identification, achieving an accuracy of 99.16% on the PlantVillage dataset. (Zhu et al., 2023) introduced EADD-YOLO, a model for accurate and efficient apple leaf disease detection model based on YOLOv5. EADD-YOLO utilized the shufflenet inverted residual blocks in the backbone and utilizing depthwise convolution to propose an efficient feature learning module in the neck. To improve detection accuracy for diseases of various sizes in different scenes, a coordinate attention module was embedded in critical locations to highlight crucial information and suppress irrelevant details. Additionally, the SIoU was used as the bounding box regression loss instead of CIoU to improve prediction box localization accuracy. Experimental results demonstrated mAP of 95.5% and 625 FPS inference on video on the apple leaf disease dataset (ALDD). Compared to other recent works on ALDD, the proposed method improved detection accuracy and speed by 12.3% and 596 FPS, respectively, with significantly fewer parameters and FLOPs. (Wang et al., 2023a) introduced Cropformer, a novel deep learning method designed for crop classification on multiple scenarios. Addressing the limitations of existing approaches that focused on extracting a single feature, Cropformer adopted a two-step classification process. In the initial step, the model undergoes a pre-training phase of self-supervised fashion to learn about crop growth features, followed by a second step involving supervised fine-tuned classification using weights derived from the first step. The study conducted comprehensive experiments on multi-scenario crop classification, covering scenarios regarding season and sample size, and transfer scenarios in 5 study areas with diverse crop types. Comparison with existing approaches revealed that the Cropformer not only achieved significantly higher accuracy in crop classification, but also demonstrated superior accuracy utilizing fewer samples. The proposed approach presents a notable advancement in addressing the challenges of multi-scenario crop classification through its unique two-step classification strategy. (Hu et al., 2023b) introduced a novel Lesion Proposal CNN based on Class-Attention called CALP-CNN designed for strawberry disease identification. The CALP-CNN employs a class response map to pinpoint the primary lesion object and suggest distinctive lesion details. Utilizing a cascading architecture, CALP-CNN concurrently addresses challenges related to complex backgrounds and the potential misclassification of similar but different instances. Experimental evaluations conducted on a self-assembled dataset of strawberry diseases attest to the effectiveness of CALP-CNN. The classification results for CALP-CNN demonstrate metrics of 92.56%, 92.55%, 91.80%, and 91.96% for accuracy, precision, recall, and F1-score, respectively. (Masood et al., 2023) introduced MaizeNet, a deep learning (DL) approach designed for the accurate identification and classification of diverse maize crop leaf diseases. Their method, an enhanced Faster-RCNN approach, employed the ResNet-50 model utilizing spatial channel attention as the underlying network for computing deep keypoints, which were subsequently localized and categorized across various classes. MaizeNet demonstrated notable effectiveness with an accuracy of 97.89% and a mAP of 94%, underscoring its efficacy in accurately locating and classifying different types of maize leaf infections.

Research on deep learning techniques for the diagnosis of cauliflower disease is noticeably lacking. Although deep learning is being used more and more in agricultural settings, especially for crop disease detection, the particular field of cauliflower diseases is still not well studied. Few researchers attempted to employ the deep learning techniques to identify cauliflower diseases. (Rajbongshi et al., 2022) introduced an online expert system based on machine vision designed for the identification of cauliflower diseases. The system processed images captured via smartphones and handheld devices then identifying them to recognize diseases and provide assistance to cauliflower farmers. The feature extraction process enabled the classification of four types of cauliflower diseases, including ‘bacterial soft,’ ‘white rust,’ ‘black rot,’ and ‘downy mildew.’ The experiment utilized 776 images, employing K-means clustering for the segmentation of disease-affected regions, followed by co-occurrence and statistical feature extraction. BayesNet, Kstar, Random Forest, LMT, BPN, and J48 classification algorithms were employed for classification of cauliflower diseases. The evaluation of these algorithms revealed that the Random Forest classifier superseded others with an accuracy approaching 89.00%. (Shakil et al., 2023) developed an agro-medical expert system for the diagnosis of cauliflower diseases. The affected portions of cauliflowers were segmented using the k-means clustering algorithm. Subsequently, 10 statistical and GLCM features were extracted from the segmented images. After selecting the top N features (where N = {5, 10}), the SMOTE technique was applied to address training datasets with varying feature quantities. Five machine learning (ML) algorithms were then utilized, and their performance was assessed for non-augmented and augmented datasets. The identical procedure was applied to both datasets, and the classifier’s performance was tested on both. Logistic regression (LR) was found as the most accurate method, achieving a 90.77% accuracy based on top 9 features in the augmented dataset. (Kanna et al., 2023) conducted experiments to assess various pre-trained DL models for the early prediction of diseases in cauliflower plants. The study focused on 3 classes of cauliflower diseases, namely Bacterial spot rot, Black rot, Downy Mildew, along with healthy cauliflower images, sourced from the VegNet dataset. Transfer learning models like EfficientNetB0, Xception, EfficientNetB1-B2-B3-B4, MobileNetV2, DenseNet201, InceptionResNetV2, and ResNet152V2, were trained and evaluated based RMS error, accuracy, precision, recall and F1-score. Notably, EfficientNetB1 demonstrated the best validation accuracy (99.90%), the smallest loss (0.16), and RMS error (0.40) during the experimentation. (Maria et al., 2022) introduced several methodologies for identifying diseases affecting cauliflower plants, comparing the effectiveness of traditional ML and TL. In their study, traditional machine learning involved image preprocessing followed by k-means clustering for image segmentation, and then the extraction of ten pertinent features. Various classification techniques were compared, with the Random Forest algorithm producing accuracy of 81.68%. Moreover, they explored CNN architectures for TL, including InceptionV3, MobileNetV2, ResNet50, and VGG16. Among these, InceptionV3 exhibited the highest accuracy at 90.08%, showcasing superior performance compared to the traditional machine learning approach. (Li et al., 2022) introduced a detection and classification model for surface defects in fresh-cut cauliflower based on a CNN with transfer learning. A dataset comprising 4,790 images of fresh-cut cauliflower, categorized into healthy, diseased, browning, and mildewed classes, was collected for the study. The authors fine-tuned the pre-trained MobileNet model to enhance both training speed and accuracy. Optimizing the model involved selecting the best configuration of hyper-parameters and freezing layers. Tests which combined VGG19, InceptionV3, and NASNetMobile, results were compared. Experimental outcomes demonstrated that, with an LR of 0.001, dropout set at 0.5, and 80 frozen layers, the MobileNet model achieved a loss value of 0.033, an accuracy of 99.27%, and an F1 score of 99.24% on the test dataset. (Abdul Malek et al., 2022) conducted research on the classification of four distinct cauliflower diseases, namely bacterial soft rot, black rot, buttoning, and white rust, utilizing several CNN models in conjunction with transfer learning. The dataset employed for this study comprised approximately 2500 images. Notably, InceptionV3 emerged as the most successful among the various CNN models investigated, achieving a remarkable test accuracy of 93.93%. This performance surpasses the outcomes observed in comparable experiments conducted in recent times.

In summary, the aforementioned works reveal significant advancements in the application of DL techniques for crop disease classification, including diverse models. These models have demonstrated high accuracy and efficiency in identifying diseases across various crops, contributing to improved agricultural practices. However, despite these notable achievements, the specific domain of cauliflower disease detection has received limited attention. While researchers have explored deep learning methods for cauliflower disease identification, there is a noticeable gap in addressing the precise localization of diseases within cauliflower images. Existing studies primarily focus on classifying diseases without providing information on the spatial distribution of symptoms within the images. This lack of emphasis on detection and localization limits the practical applicability of the models in precision agriculture, where identifying not only the presence but also the location of diseases is crucial for targeted interventions.

Motivated by this gap in the current state of cauliflower disease detection, our research aims to address the challenge of cauliflower disease detection and localization. By leveraging state-of-the-art DL techniques and drawing inspiration from successful models like YOLO, we developed a model that not only accurately classifies cauliflower diseases but also provides insights into the spatial distribution of disease symptoms within the images. This approach aligns with the broader goal of advancing precision agriculture by offering farmers a more comprehensive detection system of disease patterns in their cauliflower crops. The significance of our proposed approach lies in its potential to enhance disease management strategies by enabling farmers to pinpoint the specific locations where disease symptoms are most prevalent. This information can guide targeted interventions, such as precise application of pesticides or other treatments, minimizing resource usage and environmental impact. Ultimately, our research seeks to contribute to the development of a more robust and practical solution for cauliflower disease detection and localization, thereby addressing a critical need in the domain of precision agriculture.

## 3 Materials and methods

### 3.1 Dataset description

The VegNet dataset (Sara et al., 2022) has been meticulously curated to facilitate the effective recognition of diseases in cauliflower leaf and flower. This dataset encompasses well-organized and technically valuable images of both diseased and healthy cauliflower heads and leaves. The targeted diseases include Downy Mildew, Black Rot, and Bacterial Spot Rot. The selection of Downy Mildew, Black Rot, and Bacterial Spot Rot for this study was based on three key factors. Firstly, despite extensive searches, limited datasets covering cauliflower diseases were found, with the VegNet dataset emerging as the primary resource due to its comprehensive image coverage. Secondly, these diseases were chosen due to their prevalence and economic significance in global cauliflower crops, aligning our study with the pressing concerns of cauliflower growers and agricultural stakeholders. Lastly, each disease offers distinct visual characteristics and poses unique challenges for detection algorithms, enriching the dataset and enabling comprehensive evaluation of our proposed Cauli-Det system. While Bacterial Spot Rot is a cauliflower head disease, Downy Mildew and Black Rot mainly affect cauliflower leaves. Symptoms and disease conditions were verified by a plant pathology expert from the Bangladesh Agricultural Research Institute (BARI). The images were captured manually from the Manikganj area of Bangladesh during the period from 20 Dec to 15 Jan, 2022, when the cauliflower flowers were in full bloom and diseases were prominently observed. A Sony Cyber-Shot W-530 digital camera with a resolution of 14 MegaPixels was used to capture the images in JPEG format which were then pre-processed using Python into a standard image size of 256x256 pixels. Images in this standard format are used as the input to the YOLOv8 model. The authors of the dataset describe that the image preprocessing steps were achieved by tweaking image brightness, contrast, hue and saturation to bring forth the best visualization of disease features for the ease of detection tasks. The VegNet dataset comprises a total of 656 images, distributed among different categories based on the observed conditions. The images were split in a 70%-15%-15% ratio for training, validation and testing respectively. To ensure that the model generalizes without bias in detecting cauliflower diseases reliably, we evaluate the model’s performance on both seen data (validation dataset) and unseen data (test dataset).

**Figure 1:**
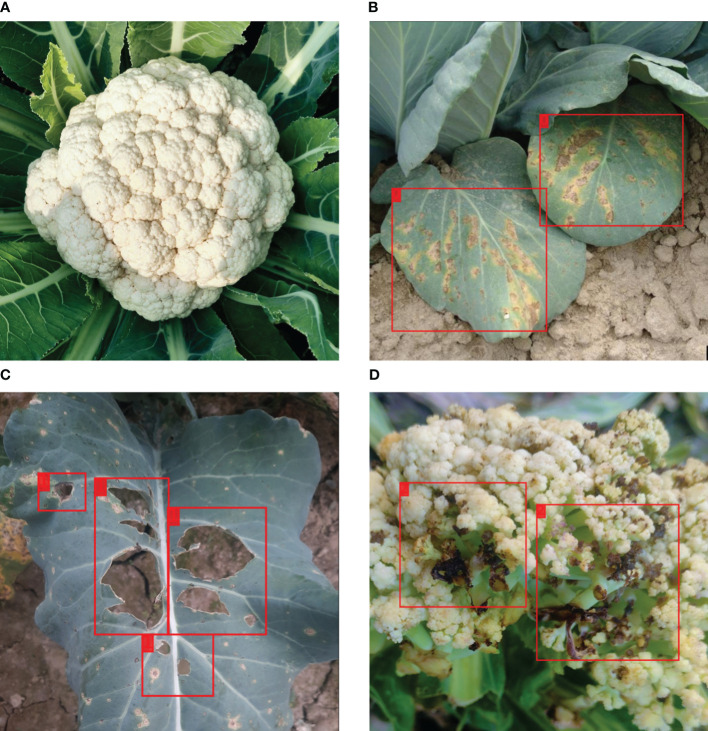
Sample images from the VegNet dataset with annotations. **(A)** Healthy cauliflower, **(B)** Downey Mildew infected cauliflower leaf, **(C)** Black Rot infected cauliflower leaf, **(D)** Bacterial Spot Rot infected cauliflower.

**Table 1** summarizes the distribution of images in the dataset per disease. **Figure 1** shows sample images from the VegNet dataset with hand drawn annotations.

**Table 1:** VegNet dataset image distribution.

Class Name	Percentage	Training	Validation	Test	Total
Downey Mildew	27.0%	125	26	26	177
Black Rot	15.2%	70	15	15	100
Bacterial Spot Rot	26.4%	121	26	26	173
Healthy	31.4%	144	31	31	206
**Total**	100%	460	98	98	656

The percentage column represents the distribution of images per class.

#### 3.1.1 Disease causes and symptoms

*Downey Mildew:* Due to a fungus called Peronospora parasitica (Muimba-Kankolongo, 2018), which causes white, yellow, or brownish patches on older leaves, accompanied by downy gray mold on the undersides are observed on leaves. Lesions and intrinsic holes facilitate penetration, releasing more spores.

Affected areas deepen in color, leading to the death of the leaf. Environmental Factors for Downey Mildew include moisture and low temperatures which favor the growth of this fungal parasite.

*Black Rot:* Xanthomonas campestris bacterial Infection causes dull, irregular yellow spots on leaf edges, progressing into V-shaped patches (Sheng et al., 2020). The wide section of the “V” is at the leaf’s border and attachment point to the plant. Symptoms may take up to a month to appear after cauliflower growth begins which renders cauliflowers unfit for sale or consumption.

*Bacterial Spot Rot:* Alternaria brassicicola (Tao et al., 2022) bacterial infection results in lesions on flower heads soaked in water form a rotting mass. Lesions often split, releasing a slimy goo that turns from first brown, then to black when exposed in the atmosphere. Transmission occurs through tools and irrigation water. Warm, moist conditions favor this disease which requires control through agricultural practices such as crop rotation, well-draining soils, and avoiding negative charges during harvest, since no chemical treatment is available.

#### 3.1.2 Dataset annotation and disease localization

The VegNet dataset comprises of image-level labels, where each whole image is labeled as either having one or no disease. Recognizing the importance of finer-grained analysis for disease management, the dataset has been enhanced through manual annotation. Using the annotation tool Makesense.ai, bounding boxes were manually drawn to localize specific regions within the images that exhibit disease symptoms. This detailed annotation approach provides a granular understanding of the spatial distribution of diseases within cauliflower plants. By precisely localizing disease-affected areas, farmers and agricultural practitioners can administer targeted treatments. This enables the application of pesticides, fungicides, or other control measures specifically to the identified regions, minimizing the use of resources and reducing environmental impact. The annotated dataset availability is included in section 6.

### 3.2 Base YOLOv8 model

**Figure 2:**
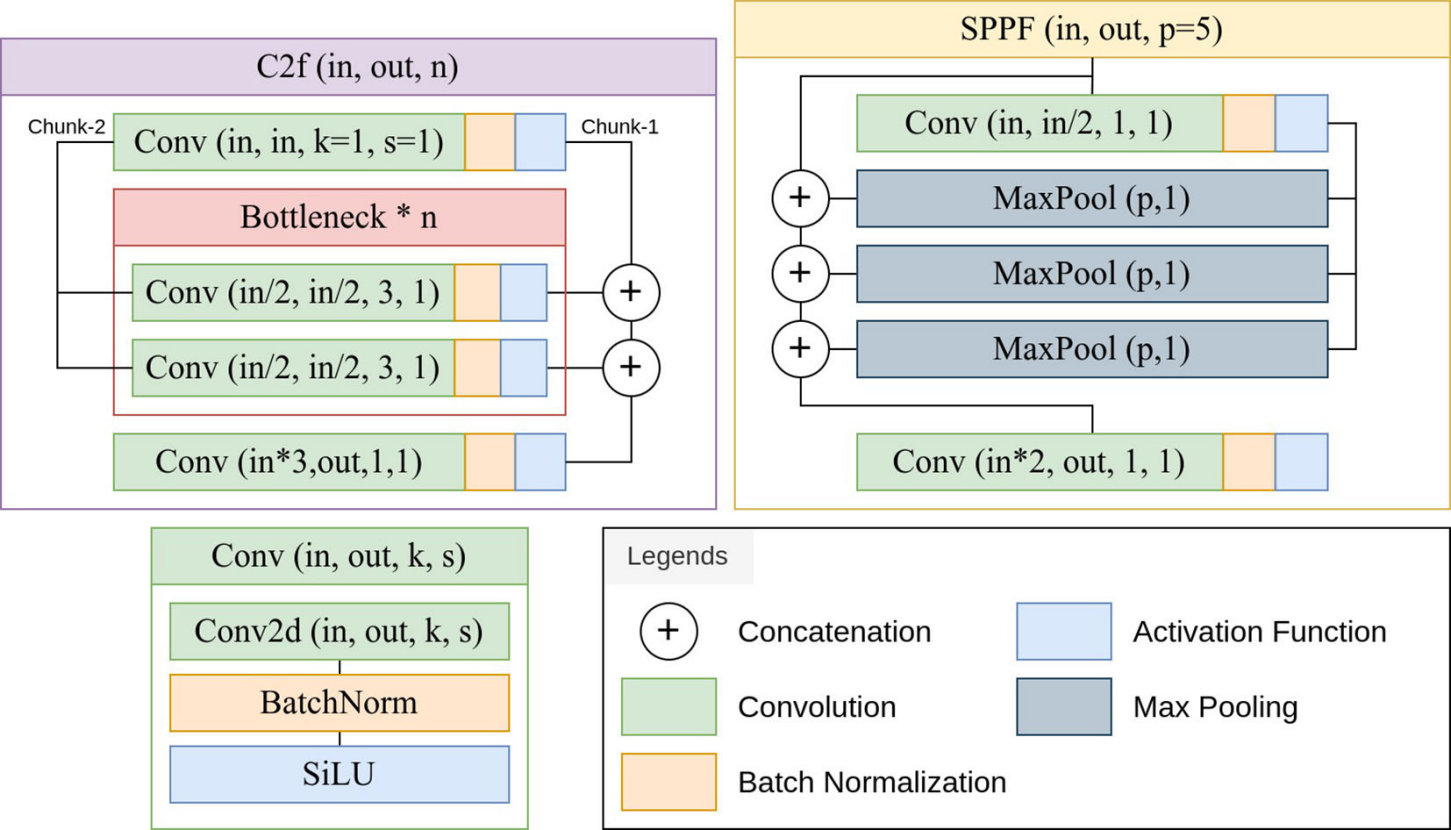
(Jocher et al., 2023) Implementation of the *Conv*, *C2f* (Cross-Stage Partial Fusion) and *SPPF* (Spatial Pyramid Pooling) blocks.

The YOLOv8 architecture begins with a series of convolutional layers with stride and kernel size configurations, followed by a batch normalization layer and an activation function. These layers reduce spatial dimensions progressively and at the same time, increases the channel number of the tensor. This is a downsampling process which facilitates the extraction of high-level features (Khan et al., 2020). A critical component of YOLOv8 is the Cross-Stage Partial Fusion module, which incorporates bottleneck structures to enhance feature representation (Wang et al., 2020). These bottlenecks consist of multiple convolutional layers with batch normalization and activation functions. A block of a convolutional layer followed by a batch normalization layer and an activation function is called a *Conv* block by the official implementation of YOLOv8 (Jocher et al., 2023). This repository also uses a faster implementation of the Cross-Stage Partial Fusion module and Spatial Pyramid Pooling module, called the *C2f* and *SPPF* module. **Figure 2** shows the implementation of the *Conv*, *C2f* and *SPPF* block using PyTorch modules. The YOLOv8 uses the spatial pyramid pooling module (*SPPF*) to capture features at multiple scales (He et al., 2015). This module utilizes max-pooling operations with different kernel sizes to aggregate contextual information. The architecture employs upsampling layers and concatenation operations to fuse features from different stages. This allows the model to refine and combine information from both high and low-level representations.

**Figure 3:**
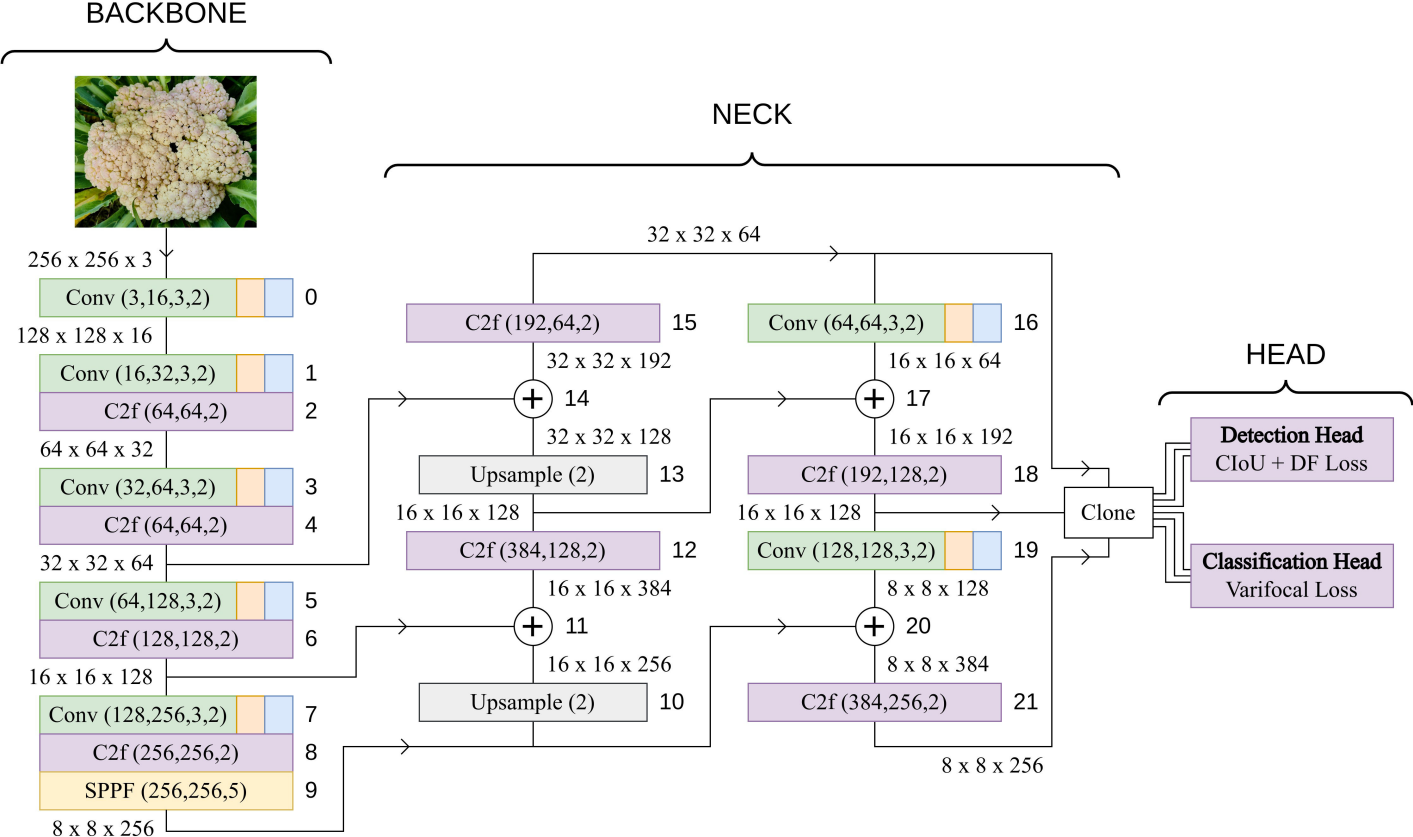
Base YOLOv8 architecture. The output dimensions of each layer is based on an input image of size 256x256.

The YOLOv8 architecture employs a multi-resolution feature fusion strategy to effectively capture both detailed and high-level information from different levels of the backbone. This process involves upsampling, concatenation, and additional convolutions to ensure that features of different resolutions are appropriately combined in the neck of the network before being fed into the detection head. The Upsampling layers increase the spatial resolution of the lower-resolution features to match that of higher-resolution features. Upsampling is performed using a specified scale factor, effectively enlarging the feature maps. *C2f* modules are inserted in the neck after each concatenation of the high and low-level features. The neck includes a subsequent convolutional layers that transform the features and adjust their channel dimensions. The output of this branch retains the original spatial resolution but gains expressive power through convolutional operations. The process of upsampling, fusion through C2f modules, and concatenation is repeated until the features from all levels of the backbone are combined. The final result is three feature maps, each containing information from different resolutions. These feature maps are then passed to the detection head for further processing and prediction of bounding box coordinates and class probabilities. The complete base YOLOv8 model architecture is visualized in **Figure 3**. The head of the model constitutes of two components:

1. Detection Head: The detection head receives three feature tensors from the neck as inputs and puts them separately through a series of *Conv* blocks and finally a convolutional layer which converts the channel number to 16 ∗ 4, where 16 is the number of Distribution Focal Loss channels and 4 is the number that signifies the attributes of the bounding box. These are [*x,y,w,h*], where *x* and *y* coordinates of the center and the width and height of the bounding box.
2. Classification Head: The classification head also receives the same three feature tensors and puts them through a series of *Conv* blocks and a final convolutional layer which converts the channel number to the number of classes, in this case 3 for the types of cauliflower diseases.

### 3.3 Modifications of YOLOv8 for cauliflower disease detection

#### 3.3.1 Adding extra *Conv* blocks to the head

**Figure 4:**
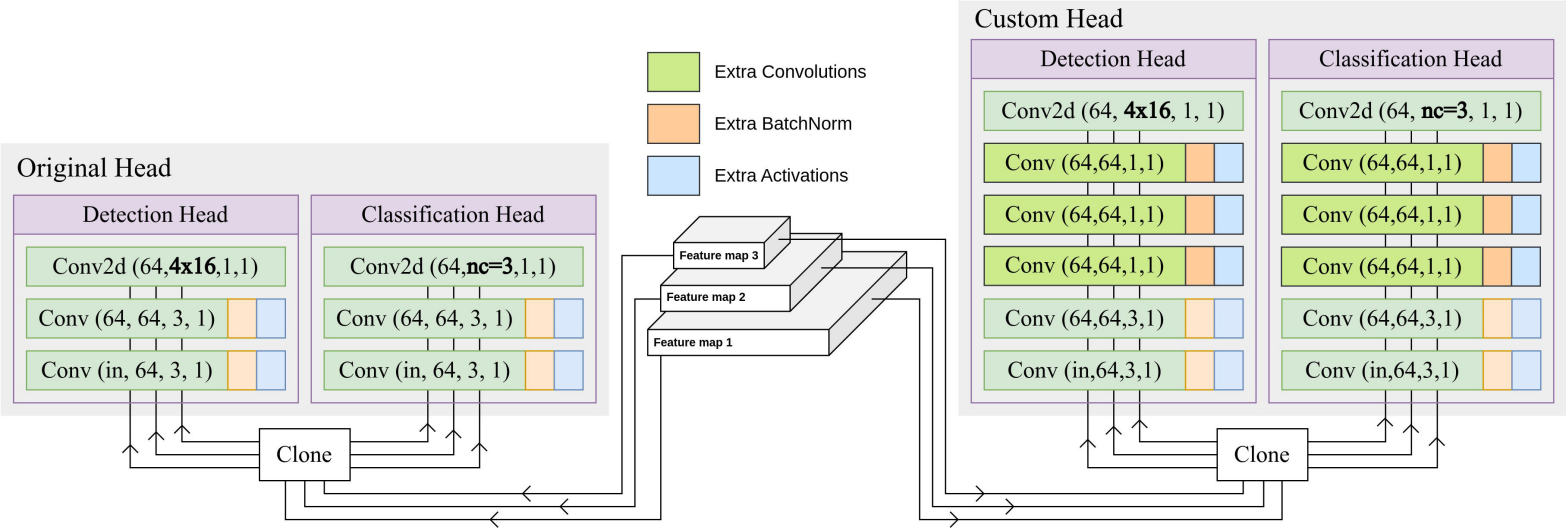
Difference between the original YOLOv8 head and the proposed custom head. 3 extra *Conv* blocks have been inserted prior to the output convolutional layer.

The original Detection and Classification head of the YOLOv8 model utilizes *Conv* blocks with a kernel size of 3, yielding a tensor with 64 channels for each of the three feature maps. Subsequently, this tensor is transformed into the requisite channels for detection and classification output through the application of a convolutional layer. In an augmentation to the base model, 3 additional *Conv* blocks, each with a kernel size of 1, have been inserted prior to the output convolutional layer. The introduction of more *Conv* blocks facilitates an increased depth within the model architecture while not increasing the number of parameters significantly, which helps with more sophisticated processing of the feature maps. Along with the capabilities of the pre-trained model, these extra *Conv* blocks improve the model’s ability to learn the domain specific information of cauliflower diseases. **Figure 4** shows the difference between the original YOLOv8 head and the proposed custom head.

#### 3.3.2 Learning rate configuration

After adding extra *Conv* blocks, we conducted experimentation to assess the ramifications of varying learning rates across discrete sections of the network. The pre-trained YOLOv8s model comes trained on the large COCO dataset. The pre-trained weights retain the feature representation power of the COCO objects. We want to preserve that knowledge and apply it to the domain of Cauliflower Disease Detection. By varying the learning rate of different sections of the network, we can control how much of the learned parameters are preserved. To find the optimal LR configuration, modifications are established wherein the parameters of the network are bifurcated into two distinct groups, denoted as the slow group and the fast group. Learning rate for the fast group remains at default levels, while the learning rate for the slow group undergoes a reduction by a factor of 100. The sections of the network subject to this parameter split include, for instance, the Slow Backbone and Fast Neck and Head, as well as the Slow Backbone and Neck and Fast Head, among others. From the results observed, it was determined that the optimal LR configuration is to use the same LR for all sections. The details of the findings are discussed in Section 4.

#### 3.3.3 Activation function of the *Conv* blocks

YOLOv8 by default employs the Swish or Sigmoid-Weighted Linear Unit (SiLU) activation function. Through a series of systematic experiments involving the exploration of diverse activation functions, it was discerned that the utilization of the Hard swish activation function yielded a marginal yet discernible enhancement in mean Average Precision (mAP). The Swish activation function (Ramachandran et al., 2017) is defined in Equation 1.


(1) f(x)=x·σ(x)

where σ represents the logistic sigmoid function.

Hard Swish (Howard et al., 2019), a modification of Swish, is formulated in Equation 2.


(2) $$h\left(x\right)=\{\begin{array}{ll} 0 & \text{if }x\leq -3,\\ x & \text{if }x\geq +3,\\ x\cdot \left(x+3\right)/6 & \text{otherwise} \end{array}$$

**Figure 5:**
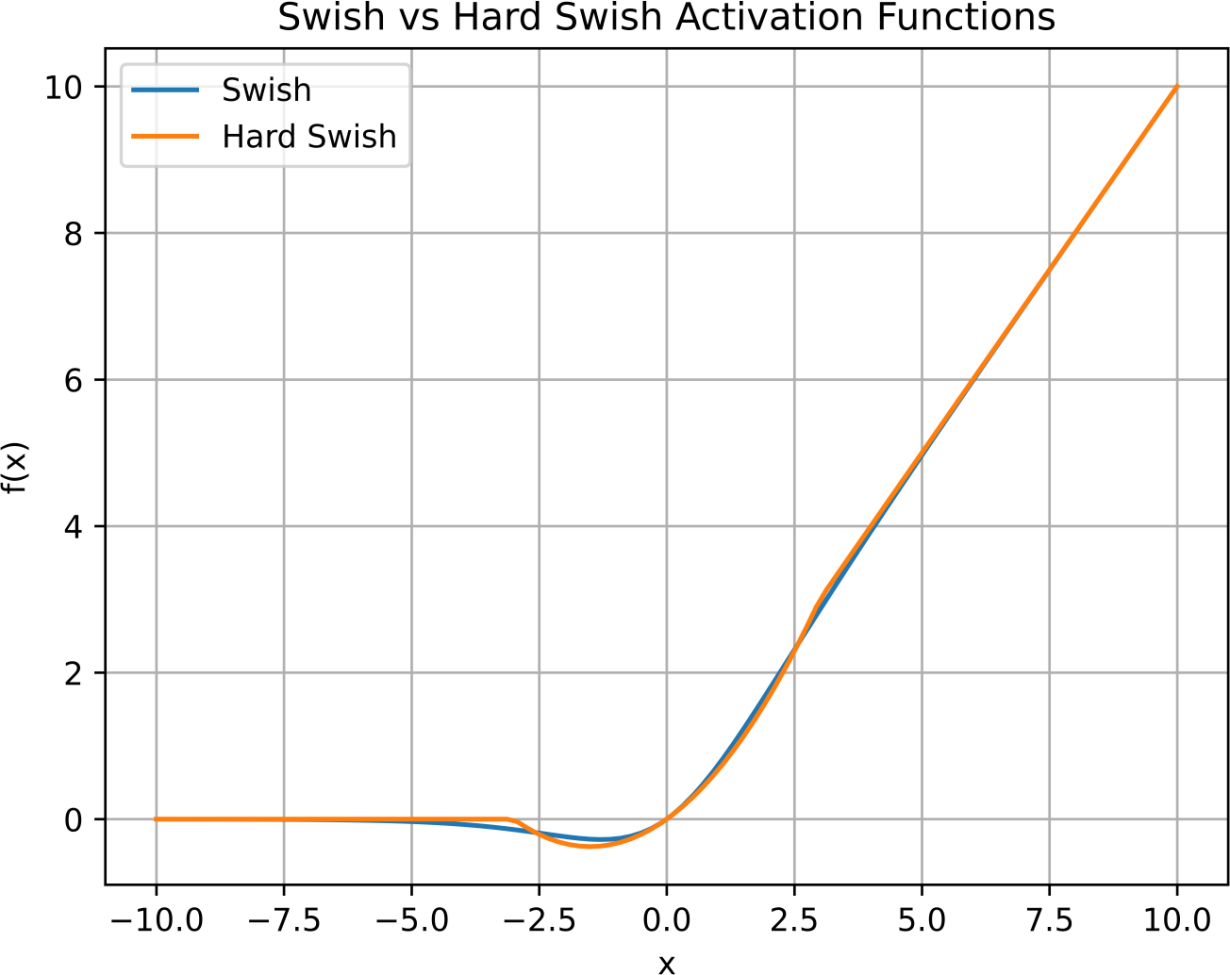
Swish and Hard Swish activation function shapes. While being very similar in output, Hard Swish is more computationally efficient.

While Swish and Hard Swish share a common foundation, the key disparity lies in the non-linear component. Swish incorporates a smooth sigmoid function, whereas Hard Swish introduces a clipped linear function. This discrepancy while not resulting in distinct shapes, provides an advantage. Swish exhibits stronger non-linear characteristics, but the computational cost associated with its smooth sigmoid component slows down training speed. Conversely, Hard Swish provides a compromise by maintaining non-linearity with a simpler clipped linear operation, leading to improved efficiency without sacrificing performance. **Figure 5** shows the Swish and Hard Swish activation functions shapes.

### 3.4 Experimental setup

#### 3.4.1 Hardware and software specifications

The source code for our experiments was built upon the Ultralytics YOLOv8 repository (Jocher et al., 2023) which was modified to compare different modification configurations. The software and hardware specifications are described in **Table 2**.

**Table 2:** Hardware and software specifications.

Hardware Setup		Software Setup	
CPU	Intel(R) Xeon(R)	Programming Language	Python 3.10.2
System Memory	12.7 GB	Deep Learning Framework	PyTorch 2.1.0
GPU	Tesla T4	GPU Support	CUDA 11.1
GPU Memory	16 GB		

#### 3.4.2 Hyperparameters

The default hyperparameters for our experiments are described in **Table 3**. These parameters hold true for all experiments unless stated otherwise.

**Table 3:** Default hyperparameters for training.

Hyperparameter	Description	Default Value
*epochs*	Number of epochs	200
*patience*	Epochs to wait for early stopping	50
*batch*	Batch size	32
*workers*	Concurrent Threads	16
*imgsz*	Resize image to	256x256 pixels
*pretrained*	Use Pre-Trained weights	True
*optimizer*	Optimizer	AdamW
*freeze*	Disable weight update for these layers	None
*lr0*	Initial Learning rate	0.001429
*momentum*	Learning Rate momentum	0.9
*box*	Box loss gain	7.5
*cls*	Class loss gain	0.5
*dfl*	DFL loss gain	1.5

#### 3.4.3 Loss functions

YOLOv8 implements 3 loss functions, all of which are summed together to form the total loss which is then passed to the optimizer. The three losses are:

• Varifocal Loss: Varifocal Loss is a loss function to train a dense object detector and predict the IoU Aware Classification Score (IACS), inspired by focal loss (Lin et al., 2017) which is used as the classification loss. Defined in Equation 3.


(3) $$\text{VFL}\left(p,q\right)=\{\begin{array}{ll} -q\times \left(q\text{log }\left(p\right)+\left(1-q\right)\times \text{log }\left(1-p\right)\right) & \text{if }q>0,\\ -\alpha p^{\gamma }\text{log }\left(1-p\right) & \text{if }q=0 \end{array}$$

where *p* is the predicted IACS and *q* is the target IoU Score and *α* is the weight coefficient.

• CIoU Loss: Complete IoU Loss, which is used as the first part of the regression loss. Defined in Equation 4.


(4) $$\text{CIoU}=1-\text{IoU}+\frac{\rho^{2}\left(b,b^{gt}\right)}{c^{2}}+\alpha \upsilon$$

where, *ρ* is the distance between the predicted bounding box and the correct bounding box *b* and *b^gt^* represent the center point of the two bounding boxes *c* is the diagonal distance of the closure area of the boxes *υ* measures the consistency of the relative proportion of the boxes.

• DFL: Distribution Focal Loss which is the second part of the regression loss, is calculated using the general distributions of bounding boxes to force the networks to learn the probabilities of values close to the target coordinates. Defined in Equation 5.


(5) $$\text{DFL}=-\left(\text{log}\left(S_{i}\right)\times \left(y_{i+1}-y\right)+\text{log}\left(S_{i+1}\right)\times \left(y-y_{i}\right)\right)$$

where, *S_i_* and *S_i_*_+1_ represent the scores or probabilities assigned to adjacent classes or categories. *y_i_* and *y_i_*_+1_ represent ground truth labels or true probabilities associated with the classes *i* and *i* + 1 respectively

#### 3.4.4 Evaluation metrics

Evaluation metrics are crucial for assessing the performance of object detection models such as YOLO. Evaluating our proposed model’s disease detection capabilities, and its performance requires a set of metrics that can quantify its accuracy and efficiency. We used the following evaluation metrics for our experiments:

• Intersection over Union (IoU): IoU measures the spatial overlap between the predicted bounding boxes (BB_pred_) and correct bounding boxes (BB_gt_). IoU is calculated as in Equation 6.


(6) $$\text{IoU}=\frac{{\text{BB}}_{\text{pred}}\cap {\text{BB}}_{\text{gt}}}{{\text{BB}}_{\text{pred}}\cup {\text{BB}}_{\text{gt}}}$$

• Precision: Precision calculates the ratio of true positives within all positive predictions, evaluating the model’s capability to avoid false positives. Precision is defined as in Equation 7.


(7) $$\text{Precision}=\frac{\text{TP}}{\text{TP}+\text{FP}}$$

• Recall: Recall calculates the proportion of true positives among all ground truth objects, evaluating the model’s ability to identify all instances of objects in the dataset. Recall is defined as in Equation 8.


(8) $$\text{Recall}=\frac{\text{TP}}{\text{TP}+\text{FN}}$$

• Average Precision (AP): AP is calculating precision-recall curves for different confidence thresholds and then computing the area under the curve (AUC). Average Precision provides a single scalar value that summarizes the model’s performance across different precision-recall trade-offs for a class.

• Mean Average Precision (mAP): It is computed by averaging the AP values across all classes. When nc is the number of classes, mAP is defined as in Equation 9.


(9) $$\text{mAP}=\frac{1}{\mathrm{nc}}\sum_{c=1}^{\text{nc}}\text{AP}\left(c\right)$$

Two different mAP values are calculated for all experiments: mAP50 (mAP is calculated with an IoU threshold of 0.5) and mAP50-95 (mAP calculated at varying IoU thresholds ranging from 0.5 to 0.95).

## 4 Result analysis

The Mean Average Precision (mAP) serves as a comprehensive metric to encapsulate the performance of object detection models, providing a singular value that reflects both precision and recall. In the context of cauliflower disease detection, the use of mAP is particularly pertinent due to its ability to gauge the model’s proficiency in identifying and localizing cauliflower disease instances in an image. The mAP metric synthesizes precision-recall curves across various thresholds, offering a concise representation of the model’s overall effectiveness. This is why we used mAP to assess the performance of baseline models as well as different modification configurations of YOLOv8 in order to find the best model for the problem of cauliflower disease detection.

### 4.1 Performance comparison of baseline models

To find the best baseline model for the problem of cauliflower disease detection, we compared the performance of YOLOv7 (Wang et al., 2023b), along with YOLOv8’s nano, small, medium, large and extra large models. The results of these experiments are shown in **Table 4**. All models were pretrained on the COCO object detection dataset (Lin et al., 2014).

**Table 4:** Performance and parameters of base YOLOv7 and YOLOv8 models on the validation dataset and test dataset.

Model	Precision val | test	Recall val | test	mAP50 val | test	mAP50-95 val | test	Parameters
YOLOv7	**0.964** | **0.978**	**0.941** | **0.889**	**0.955** | **0.926**	**0.716** | 0.718	37.21 M
YOLOn8n	0.898 | 0.910	0.809 | 0.828	0.844 | 0.821	0.562 | 0.577	3.01 M
YOLOv8s	0.932 | 0.914	0.817 | 0.832	0.886 | 0.841	0.599 | 0.661	11.14 M
YOLOv8m	0.919 | 0.912	0.865 | 0.868	0.916 | 0.916	0.702 | 0.721	25.86 M
YOLOv8l	0.929 | 0.904	0.870 | 0.875	0.924 | 0.915	0.689 | 0.711	43.63 M
YOLOv8x	0.938 | 0.918	0.872 | 0.847	0.923 | 0.910	0.694 | **0.723**	68.16 M

Bold values signify the value of the best performing base model under each metric.

**Figure 6:**
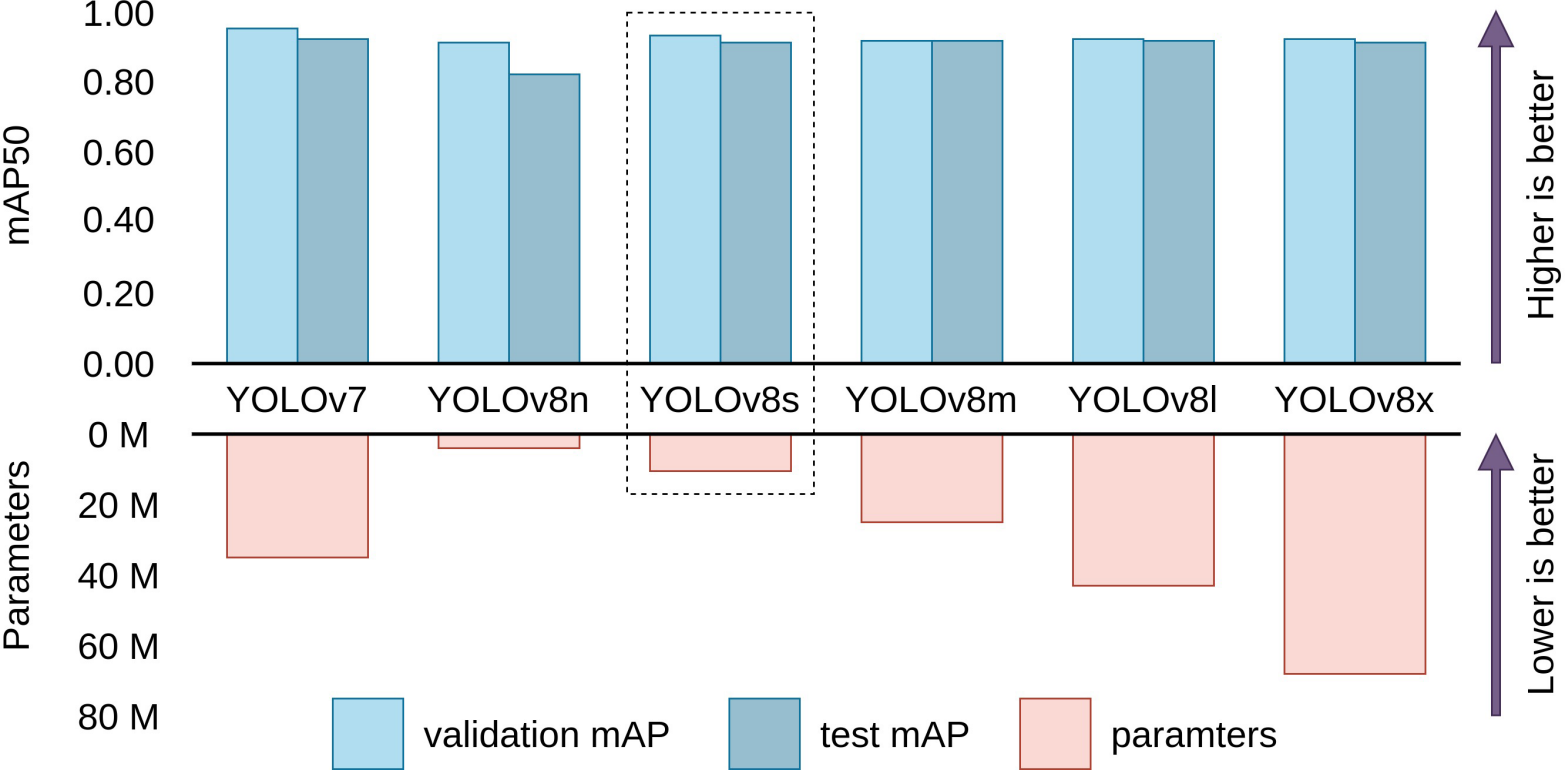
Comparison between performance and parameters of baseline models. YOLOv8s is evidently the most efficient model with similar validation and test performance as the other larger models. (Scales are relative).

**Figure 6** visualizes the performance and parameters counts between mAP and parameters of baseline models. We observe that, while YOLOv7 has the highest validation and test mAP (95.5% and 92.6%) followed closely by YOLOv8s (92.9% and 91.0%), YOLOv7 has more than thrice the parameter count (37.21 million) than YOLOv8s (11.14 million). Other larger sizes of YOLOv8 are observed to not perform up to the expectation that comes with their higher parameter counts. This phenomenon can be attributed to redundant network depth. Smaller models like the YOLOv8s has enough depth to capture and model the features of cauliflower disease images, resulting in bigger models unable to perform better than YOLOv8s. Increased depth also results in these models being unfit to be employed on lower-end devices for practical applications like video inference. On the other hand, YOLOv8s has the lowest validation and test mAP due its small size. It is thus concluded that YOLOv8s has the best balance of performance and parameter count, judging by the law of diminishing return. Therefore we chose YOLOv8s for further modification and evaluation with a goal of improving the performance while not increasing the parameter count significantly.

### 4.2 Performance comparison of modified YOLOv8s

#### 4.2.1 Comparison of head configurations

The primary focus was on enhancing the performance of YOLOv8s without significantly increasing the parameter count. The approach employed involved the addition of extra *Conv* blocks to both the detection and classification heads of YOLOv8s. The goal of our experimentation was to discern the impact of augmenting the head on the performance of YOLOv8s. The configurations employed involved adding 1, 2, 3, 4, and 5 extra *Conv* blocks to determine which is the best performing. **Table 5** shows the results of adding different numbers of *Conv* blocks to the head of YOLOv8s.

**Table 5:** Performance and parameter count of Base YOLOv8s and YOLOv8s with extra *Conv* blocks on the validation and test dataset.

Model	Precision val | test	Recall val | test	mAP50 val | test	mAP50-95 val | test	Parameters
YOLOv8s	0.932 | 0.914	0.817 | 0.832	0.886 | 0.841	0.599 | 0.661	11.14 M
YOLOv8s+Conv	0.918 | **0.955**	0.657 | 0.837	0.715 | 0.905	0.545 | 0.688	11.20 M
YOLOv8s+Conv2	**0.943** | 0.901	0.829 | 0.852	0.894 | 0.894	0.670 | 0.694	11.26 M
YOLOv8s+Conv3	0.899 | 0.931	0.846 | 0.829	0.904 | **0.906**	**0.674** | **0.694**	11.32 M
YOLOv8s+Conv4	0.931 | 0.936	**0.852** | **0.859**	**0.905** | 0.903	0.657 | 0.686	11.38 M
YOLOv8s+Conv5	0.937 | 0.946	0.833 | 0.857	0.905 | 0.904	0.662 | 0.688	11.45 M

Bold values signify the value of the best performing model configuration under each metric.

**Figure 7:**
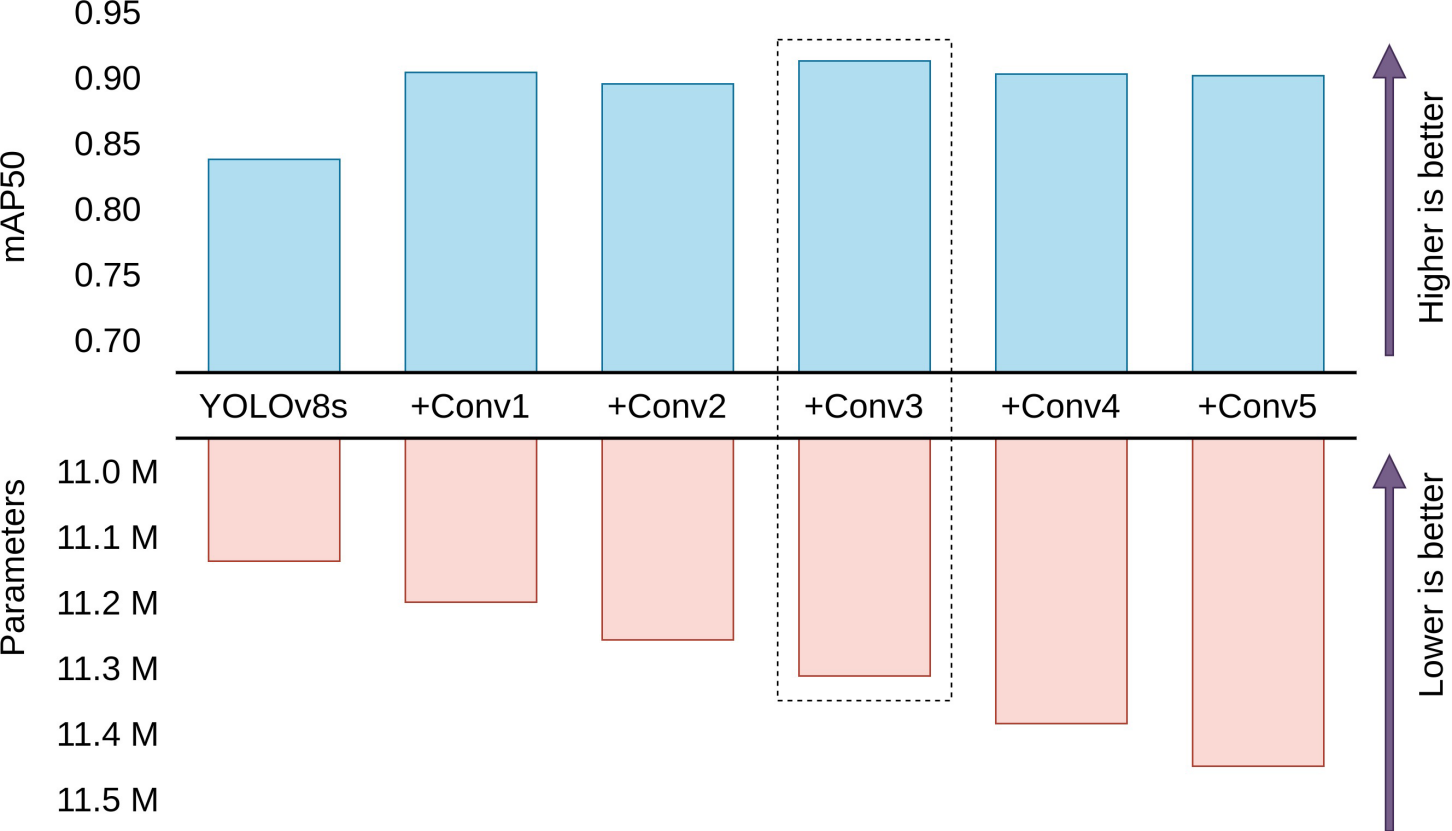
Comparison between performance and parameters of different head configurations. YOLOv8s with 3 extra *Conv* blocks is found to be the best model with the highest mean average precision for the test dataset. (Scales are relative).

**Figure 7** visualizes the performance and cost tradeoffs of adding different numbers of *Conv* blocks. The analysis of the results demonstrates that adding 3 extra *Conv* blocks yields the best mean average precision on the test dataset. Intriguingly, further augmentation to 5 *Conv* blocks do not bring a significant improvement in performance. The diminishing returns observed with the addition of more than 3 *Conv* blocks can be attributed to a phenomenon of diminishing feature discriminability. While the initial addition of extra convolutional blocks contributes to the model’s ability to capture and learn complex features, the excessive introduction of these blocks can lead to overfitting or redundant feature extraction. As a result, the model may become overly specialized on the training data, impairing its generalization ability on unseen data.

#### 4.2.2 Comparison of different LR optimization

Following the incorporation of 3 additional *Conv* blocks that demonstrated improved model performance, we further refined the training strategy by exploring the impact of different learning rates across distinct sections of the network. Given the utilization of a pre-trained model as the base, the objective is to fine-tune the learning rates strategically to balance the preservation of learned parameters in the original layers with accelerated learning in the newly added components. The experimentation encompassed six distinct configurations:

- Default: All sections of the network share a uniform learning rate.
- Freeze–Non–Extra–Conv: All layers except the newly added *Conv* blocks are frozen during training.
- Freeze–Back: The backbone is frozen, while the neck and head layers remain trainable with the same learning rate.
- Fast–Extra–Conv: Original layers of the model receive a learning rate reduced by a factor of 100, while the extra *Conv* blocks maintain their original learning rate.
- Fast–Head: Both the backbone and neck undergo a 100x reduction in learning rate, while the head retains the original learning rate.
- Fast–Head–Neck: Only the backbone experiences a 100x reduction in learning rate, with the neck and head maintaining their original learning rates.

**Table 6** provides the results of these experiments. Surprisingly, the experimentation revealed that freezing either the entire backbone or all layers except the newly added layers resulted in a significant decline in performance. This outcome was attributed to the restrictive nature of preventing the adjustment of layer weights, compelling the model to rely solely on the pretrained feature extraction. This approach proved suboptimal for detecting cauliflower diseases, as the model struggled to adapt its pre-learned features to the specific nuances of this domain.

**Table 6:** Comparison of different LR configurations on the validation dataset and test dataset.

Configuration	Precision val | test	Recall val | test	mAP50 val | test	mAP50-95 val | test
Default (YOLOv8s+Conv3)	0.899 | 0.931	**0.846** | 0.829	**0.904** | **0.906**	**0.674** | **0.694**
Freeze–Non–Extra–Conv	0.266 | 0.299	0.308 | 0.352	0.226 | 0.272	0.110 | 0.136
Freeze–Back	0.560 | 0.566	0.363 | 0.461	0.417 | 0.511	0.216 | 0.280
Fast–Extra–Conv	0.883 | 0.906	0.687 | 0.765	0.811 | 0.839	0.545 | 0.603
Fast–Head	0.853 | 0.920	0.750 | 0.778	0.830 | 0.846	0.549 | 0.615
Fast–Head–Neck	**0.938** | **0.955**	0.830 | **0.837**	0.895 | 0.905	0.659 | 0.688

Bold values signify the value of the best performing LR configuration under each metric.

Moreover, the results indicated that employing a slower learning rate for any section, even with the intention of preserving pretrained knowledge, led to a small but noticeable drop in performance. Consequently, the decision was made to allow all layers to freely adjust their weights using the default learning rate. This approach yielded the best LR configuration, striking a balance between leveraging the knowledge encoded in the pretrained layers and enabling the model to fine-tune its parameters for improved performance in the targeted cauliflower disease detection domain.

#### 4.2.3 Comparison of different activation functions

Building upon the optimal configuration identified in the previous experiments, where 3 extra *Conv* blocks significantly improved overall performance in YOLOv8s with all layers sharing the same learning rate, we delved into the impact of altering the default activation function. The original YOLOv8 utilizes SiLU, also known as the swish activation function, as the default choice throughout the network. In this subsequent experimentation, we systematically replaced SiLU with alternative activation functions, such as ReLU, Leaky ReLU, Tanh, and Hard Swish, to discern their effects on model performance. The efficacy of Hard Swish can be attributed to its unique characteristics, combining non-linearity with bounded activation values. This enables the model to capture complex patterns while mitigating issues related to vanishing gradients or over-amplification of certain features. **Table 7** summarizes the results of this experiment. Hard Swish was found to be the overall best performing activation function, which is also more computationally efficient than the default Swish since Hard Swish doesn’t have to calculate a non-linear function like the sigmoid and has a linear mathematical definition which is better for reducing training and inference time.

**Table 7:** Performance summary of YOLOv8s with additional 3 Conv blocks and alternative activation functions on the validation dataset.

Model	Precision val | test	Recall val | test	mAP50 val | test	mAP50-95 val | test
YOLOv8s+Conv3+SiLU	0.899 | 0.931	0.846 | 0.829	0.904 | 0.906	0.674 | 0.694
YOLOv8s+Conv3+ReLU	0.905 | 0.906	0.815 | 0.829	0.878 | 0.875	0.626 | 0.668
YOLOv8s+Conv3+LeakyReLU	0.915 | 0.942	0.834 | **0.840**	0.892 | 0.900	0.642 | 0.677
YOLOv8s+Conv3+Tanh	0.822 | 0.906	0.623 | 0.702	0.726 | 0.771	0.434 | 0.502
YOLOv8s+Conv3+Hard Swish	**0.919** | **0.932**	**0.851** | 0.826	**0.920** | **0.911**	**0.677** | **0.701**

Bold values signify the value of the best performing activation function configuration under each metric.

#### 4.2.4 Evaluation of the proposed model

The proposed model, derived from the extensive experimentation and fine-tuning process, was evaluated on the validation and test dataset to assess its performance comprehensively. **Supplementary Figure 1** displays the normalized confusion matrices, **Supplementary Figure 2** displays the precision-confidence curves, **Supplementary Figure 3** displays the recall-confidence curve, **Supplementary Figure 4** displays the F1-confidence curve, **Supplementary Figure 5** displays the precision recall curves of our proposed model on the validation and test dataset respectively. **Tables 8**, **9** summarizes the performance of the proposed model on the validation and test dataset by class respectively. **Supplementary Figure 6** displays the training and validation loss along with precision, recall, mAP50 and mAP50-95 progression over the training period.

**Table 8:** Performance summary of the proposed model on the validation dataset by class.

Class	Images	Instances	Prec	Rec	AP50	AP50-95
All	98	332	0.919	0.851	0.920	0.677
Downey Mildew	26	77	0.919	0.844	0.941	0.703
Black Rot	15	215	0.852	0.758	0.845	0.532
Bacterial Spot Rot	26	40	0.987	0.950	0.974	0.797
Healthy	31	0	–	–	–	–

**Table 9:** Performance summary of the proposed model on the test dataset by class.

Class	Images	Instances	Prec	Rec	AP50	AP50-95
All	98	309	0.932	0.826	0.911	0.701
Downey Mildew	26	50	0.901	0.840	0.925	0.686
Black Rot	15	225	0.926	0.667	0.826	0.552
Bacterial Spot Rot	26	34	0.968	0.971	0.983	0.864
Healthy	31	0	–	–	–	–

## 5 Discussion

The conducted experiments focused on enhancing the YOLOv8 model for cauliflower disease detection, aiming for improved performance without significantly increasing parameters. Initial analysis showed that YOLOv7 and YOLOv8l had high mean average precision (mAP) but were impractical for lower-end devices due to large parameter counts. YOLOv8s, with a balanced trade-off between performance and parameters, was selected. Modifications involved adding extra *Conv* blocks to detection and classification heads was explored. Results indicated that incorporating three additional blocks yielded the best performance, with further augmentation leading to diminishing returns likely due to overfitting and redundant feature extraction. The study refined the training strategy, revealing that allowing all layers to freely adjust weights using the default learning rate achieved the optimal configuration. Freezing layers resulted in a decline in performance. The impact of activation functions was explored, with the default Swish emerging as the best-performing choice. In summary, systematic exploration led to an optimized YOLOv8s configuration, with three extra *Conv* blocks, balanced learning rates, and Swish as the activation function, demonstrating superior performance in cauliflower disease detection. However, limited disease variety in the dataset is a major limitation of this research which restricts the generalization scope of Cauli-Det, which included only three types of cauliflower diseases. Given the diverse range of diseases that can affect cauliflower leaves, flowers, and stems, the model’s effectiveness may be limited when confronted with other diseases not represented in the dataset. The study focused on optimizing the YOLOv8s model for deployment on lower-end devices. However, the performance evaluation may not fully capture the nuances of diverse hardware configurations. The model’s efficiency and accuracy could vary on different devices, and further research may be required to fine-tune the model for optimal performance across a wider range of computing resources. The evaluation of the model’s performance was primarily conducted based on offline analysis of collected data. Real-time evaluation, crucial for practical deployment in agriculture, was not explicitly addressed in this research. The model’s responsiveness to dynamic changes in the field, such as disease progression or plant growth, remains an unexplored aspect.

## 6 Conclusion

This research offers a tailored YOLOv8s model specifically designed for detecting prevalent cauliflower diseases, addressing unique challenges in disease identification. Additionally, it provides a comprehensive evaluation of base YOLOv8 models on cauliflower disease datasets, highlighting baseline performance and paving the way for systematic model modifications. By systematically applying adjustments to enhance detection accuracy and average precision, the study offers valuable insights into improving the model’s ability to classify cauliflower diseases effectively. Furthermore, the paper contributes to the research community by providing open access to an annotated dataset and the proposed model, fostering reproducibility and facilitating further advancements in computer vision applied to agriculture. We identified the limitations of the proposed model and for future research approaches that may build upon this work to build a better and more capable cauliflower disease detection approach, we plan to address more diseases than the three that were present in the dataset used in this work (Downey Mildew, Black Rot, and Bacterial Soft Rot). We also believe that the success of any disease detection model will be dependent on its real-world applicability, which will rely on demonstrating the models performance on lower end devices which are most likely to be available on the hands of crop farmers. Additionally, another challenge is the evaluation of these techniques in real-time disease detection, for which video datasets will be required. In a real-time system it is crucial to demonstrate the model’s responsiveness to dynamic changes in the field. Drones may be employed to capture live video feed from cauliflower fields which then may be streamed into a model with fast inference to demonstrate the model’s real-time disease detection capabilities. Some cauliflower diseases are more prevalent than others depending on region and climate. Conducting research on these diverse set of circumstances can help other researchers on making informed choices when developing tailored disease detection models that are fit for addressing disease management problems according to region, climate and the specific needs of cauliflower plantations. Lastly, We believe that the proposed model will be a valuable addition to the field of disease detection for the domain of precision agriculture.

## Data availability statement

The original contributions presented in the study are included in the article/**Supplementary Material**. Further inquiries can be directed to the corresponding author. The source code for the modified YOLOv8 network is available at: https://github.com/manchitro/vegnet-yolov8. Best weights are available at: https://github.com/manchitro/vegnet-yolov8/releases.

## Author contributions

MU: Conceptualization, Writing – original draft. MKAM: Data curation, Methodology, Writing – original draft. AP: Formal analysis, Validation, Writing – original draft. MFM: Writing – review & editing. MS: Supervision, Validation, Writing – review & editing. SA: Funding acquisition, Visualization, Writing – review & editing. DC: Formal analysis, Validation, Writing – review & editing.
