# Supplementary material for: Cauli-Det: enhancing cauliflower disease detection with modified YOLOv8
Source: Front Plant Sci. 2024 Apr 18;15:1373590. doi: 10.3389/fpls.2024.1373590 (PMC11063243; doi:10.3389/fpls.2024.1373590)
Supplement: Supplementary file 1 [file DataSheet_1.pdf]

Supplementary Figures

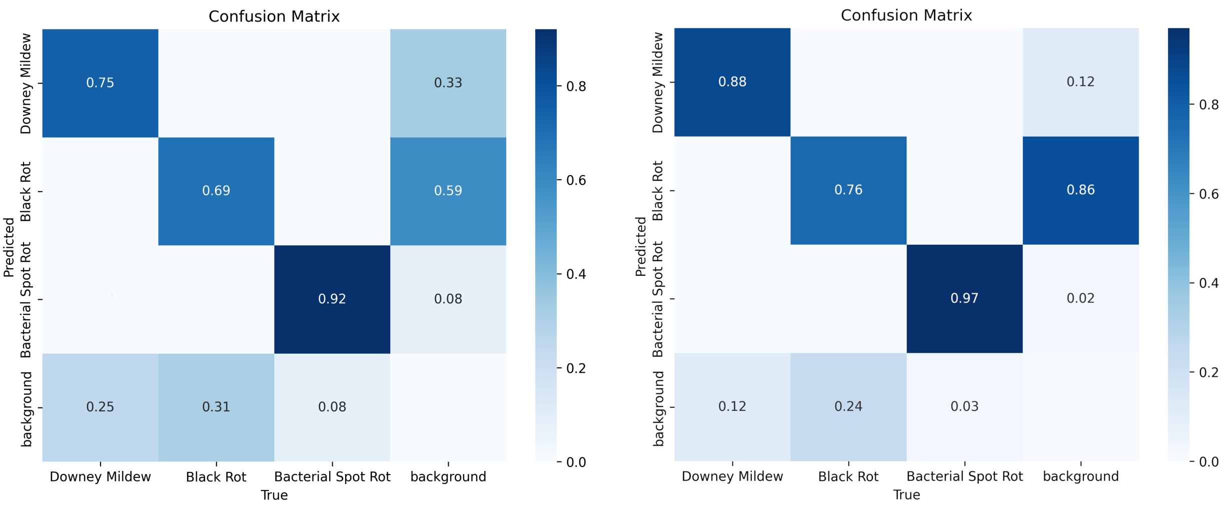

(A) (B)

Figure S1. Normalized Confusion matrix of the proposed model on the validation dataset (A) and test dataset (B).

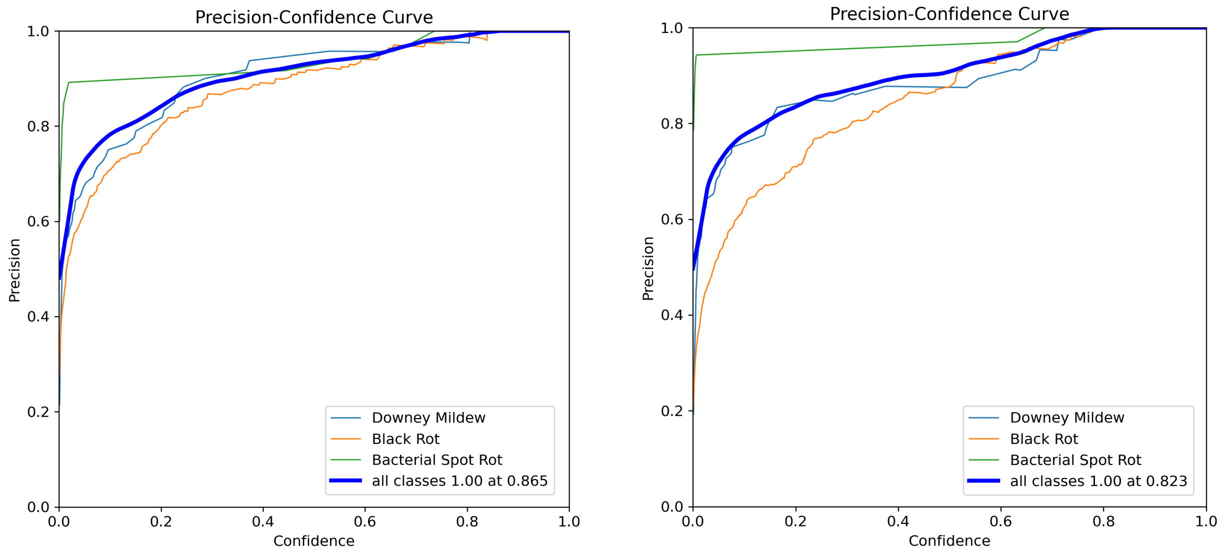

(A) (B)

Figure S2. Precision-Confidence graph of the proposed model on the validation dataset (A) and test dataset (B).

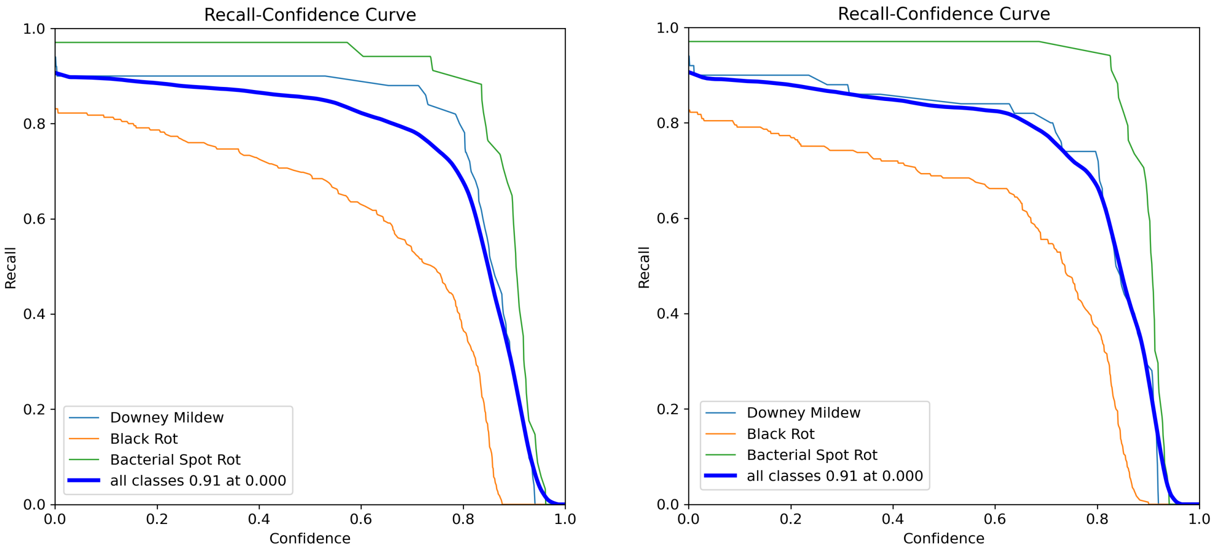

(A) (B)

Figure S3. Recall-Confidence graph of the proposed model on the validation dataset (A) and test dataset (B).

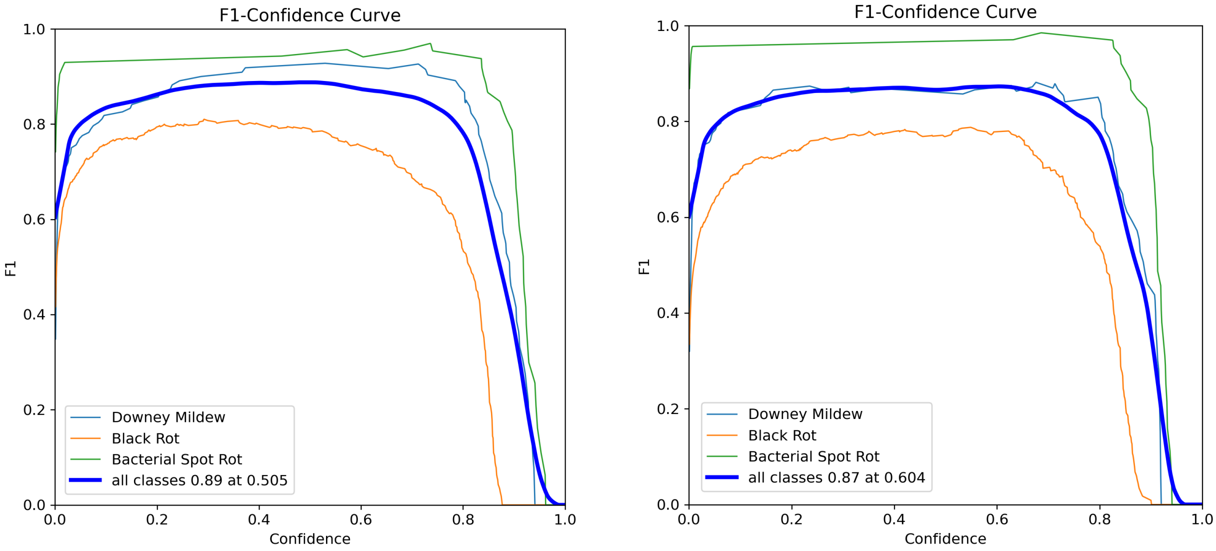

(A) (B)

Figure S4. F1-Confidence graph of the proposed model on the validation dataset (A) and test dataset (B).

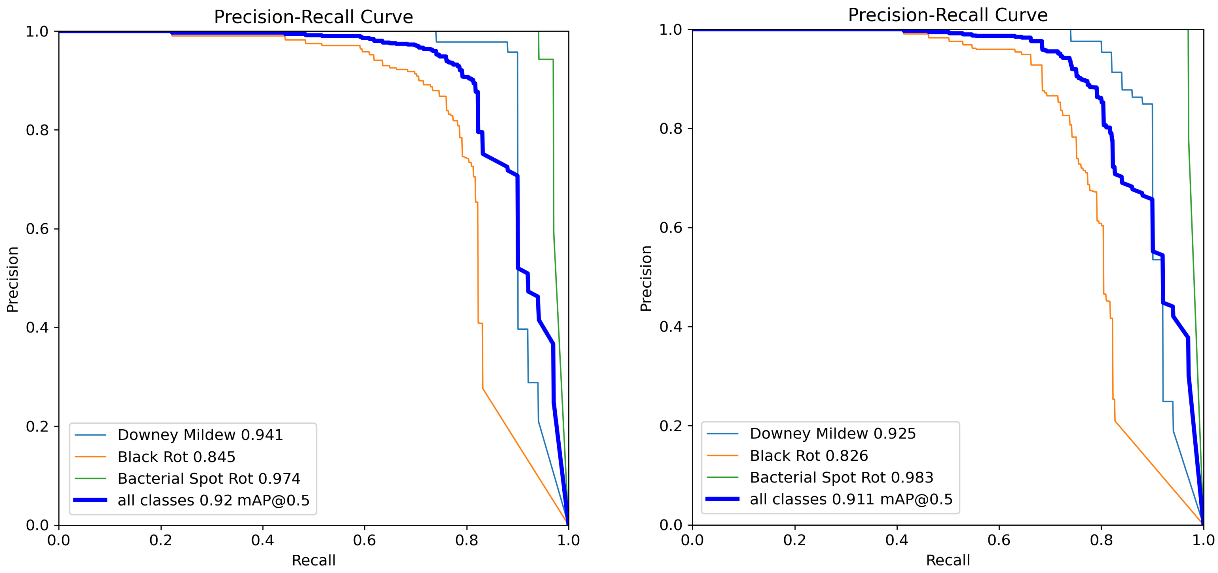

(A) (B)

Figure S5. Precision-Recall graph of the proposed model on the validation dataset (A) and test dataset (B).

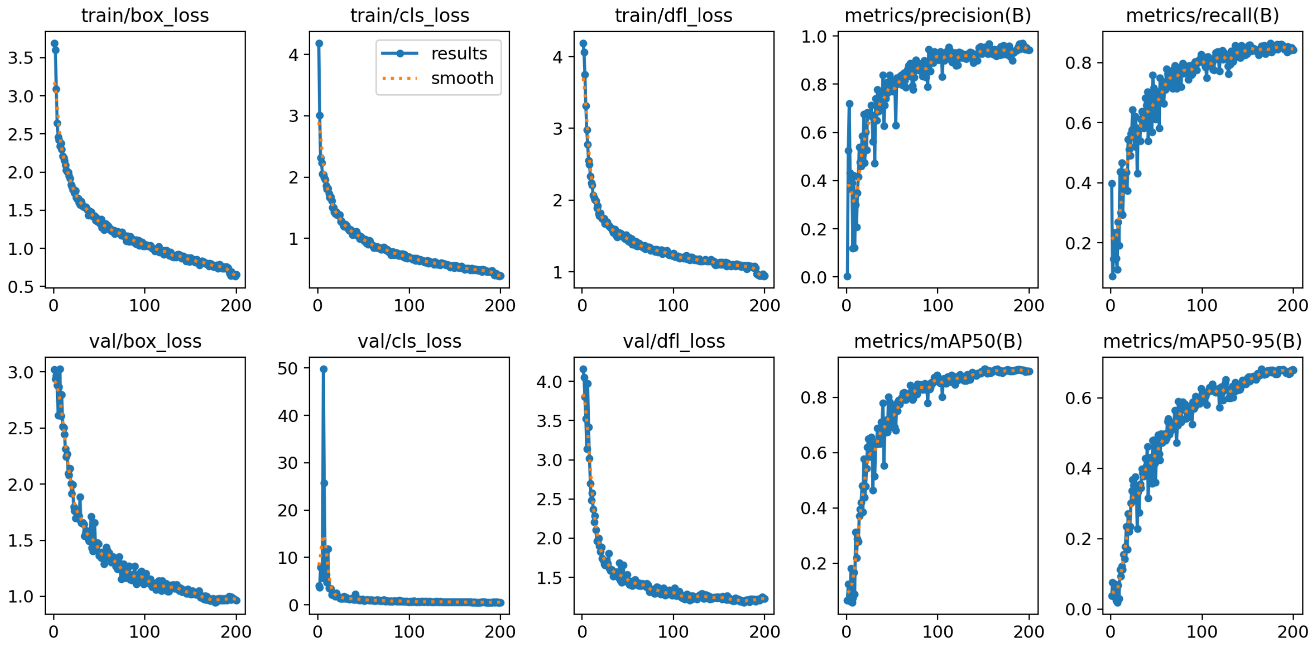

Figure S6. Performance metrics progression over training period for the best-performing model.
